# Supplementary material for: A Critical View of the Application of the APEX Software (Aqueous Photochemistry of Environmentally-Occurring Xenobiotics) to Predict Photoreaction Kinetics in Surface Freshwaters
Source: Molecules. 2019 Dec 18;25(1):9. doi: 10.3390/molecules25010009 (PMC7017383; doi:10.3390/molecules25010009)
Supplement: Supplementary file 1 [file molecules-25-00009-s001.zip › 3.2.4_gcc-4.4.0.zip!/3.2.4_gcc-4.4.0/3.2.4_gcc-4.4.0/doc/octave/html/interpreter/Packages.html]

Packages - Untitled


Next: Dynamically Linked Functions,
Previous: System Utilities,
Up: Top


---

## 35 Packages

Since Octave is Free Software users are encouraged to share their
programs amongst each other. To aid this sharing Octave supports the
installation of extra packages. The `Octave-Forge' project is a
community-maintained set of packages that can be downloaded and
installed in Octave. At the time of writing the `Octave-Forge' project
can be found on-line at http://octave.sourceforge.net, but
since the Internet is an ever-changing place this may not be true at
the time of reading. Therefore it is recommended to see the Octave
website for an updated reference.

- Installing and Removing Packages- Using Packages- Administrating Packages- Creating Packages
